# Impact of Genetic Variants of Long Noncoding RNA* Metastasis-Associated Lung Adenocarcinoma Transcript 1* on Uterine Cervical Cancer

**DOI:** 10.7150/jca.70730

**Published:** 2022-04-04

**Authors:** Yi-Hung Sun, Ying-Hsiang Chou, Hsueh-Yu Tsai, Yi-Hsuan Hsiao, Chung-Yuan Lee, Shun-Fa Yang, Ke-Hsin Ting, Po-Hui Wang

**Affiliations:** 1 Institute of Medicine, Chung Shan Medical University, Taichung, Taiwan.; 2 Department of Obstetrics and Gynecology, Chi-Mei Foundation Medical Center, Tainan, Taiwan.; 3 Department of Medical Imaging and Radiological Sciences, Chung Shan Medical University, Taichung, Taiwan.; 4 Department of Radiation Oncology, Chung Shan Medical University Hospital, Taichung, Taiwan.; 5 Department of Obstetrics and Gynecology, Chung Shan Medical University Hospital, Taichung, Taiwan.; 6 School of Medicine, Chung Shan Medical University, Taichung, Taiwan.; 7 Department of Obstetrics and Gynecology, Changhua Christian Hospital, Changhua, Taiwan.; 8 Department of Obstetrics and Gynecology, Chiayi Chang Gung Memorial Hospital Chiayi, Taiwan.; 9 Department of Nursing, Chang Gung University of Science and Technology, Chiayi Campus, Chiayi, Taiwan.; 10 Division of Cardiology, Department of Internal Medicine, Changhua Christian Hospital, Yunlin Branch, Yunlin, Taiwan.

**Keywords:** long noncoding RNA* metastasis-associated lung adenocarcinoma transcript 1*, genetic variants, uterine cervical cancer, lymph node metastasis

## Abstract

Genetic variants of long noncoding RNA* metastasis-associated lung adenocarcinoma transcript 1* (lncRNA *MALAT1*) have been reported to be associated with several cancers. Until now, no study reveals the associations between lncRNA *MALAT1* polymorphisms and cervical cancer (CC)*.* The objectives of this study were to explore the correlations among *MALAT1* polymorphisms and occurrence and clinicopathological parameters of CC, as well as patient 5 years survival in Taiwanese women. The study recruited 116 patients with cervical invasive cancer and 89 patients with cervical precancerous lesions, as well as 268 non-cancer control women. LncRNA *MALAT1* polymorphisms rs3200401, rs619586 and rs1194338 were selected and their genotypic frequencies were defined by real-time polymerase chain reaction. Our results revealed that there are no relationships between lncRNA *MALAT1* genetic variants and occurrence of CC. The independent factor among lncRNA *MALAT1* genetic variants and clinicopathological parameters were positive pelvic lymph node metastasis (p=0.001, HR: 10.94, 95% CI: 2.65-45.23). In conclusions, lncRNA *MALAT1* genetic variants are not related to occurrence and clinicopathological characteristics of CC and patient 5 years survival in Taiwanese women. Pelvic lymph node metastasis could independently predict the patient 5 years survival among various *MALAT1* polymorphisms and clinicopathological factors in CC.

## Introduction

Metastasis-associated lung adenocarcinoma transcript 1 (*MALAT1*) is a long noncoding RNA (lncRNA), which is defined as a group of RNAs more than 200 nucleotides with restricted or no coding capacity, because of lacking an intact open reading frame. But it can influence gene expression through various patterns including assembly of chromatin-modifying complex, micro RNA sponges, promotion or suppression of gene expression, promotion or repression of DNA methylation, involvement of cell migration, proliferation and invasion as well as apoptosis, and therefore may be concerned with a number of important biological activities 1-4. LncRNA *MALAT1* is an 8.5 kb lncRNA and located at chromosome 11q13, which is abundant in a variety of human cell types, presenting the highest expression in lung and pancreas cell 5, 6. *MALAT1* has been demonstrated to be abnormally regulated and exhibits prognostic significance in several cancers, including hepatocellular carcinoma, breast cancer, colorectal cancer, and gastric cancer as well as esophageal cancer 7-9. Overexpression of *MALAT1* was found in cervical cancer (CC) tissues, as compared to normal cervical tissues 10. In addition, *MALAT1* has been revealed to be significantly associated with tumor size, International Federation of Gynecology and Obstetrics Classification (FIGO) stage and lymph node metastasis of CC 10-12.

Cancer of uterine cervix is the fourth most frequent malignancy in female individuals globally, which is responsible for near 604,000 new cases in 2020 13. It has been shown that the annual incidence of uterine cervical cancer (CC) in the world was estimated to be 14.0 per 100,000 populations 14. However, in Taiwan, the age-standardized incidence rate of CC was reported to be 8.50 per 100,000 women, and ranked the eighth most frequent cancer among women in 2014 based on the data of the Health Promotion Administration of the Ministry of Health and Welfare as well as Annual Cancer Registry Report. The age-standardized mortality rate of CC was estimated to be 3.39 per 100,000 women in this year, accounting for the seventh leading cause of cancer mortality among female individuals in the country. Tumorigenesis of uterine cervix is a continuous multistep dysplasia process 15. Cervical intraepithelial neoplasia (CIN) may persist its progression into invasive cancer 16. Cervical intraepithelial neoplasia 1 (CIN 1) conforms to low-grade squamous intraepithelial lesion in cytological counterpart (LSIL; also known as low-grade CIN or mild dysplasia for histologic term), in which mitotic and immature cells only exist in the lower third of the epithelium; whereas CIN 2 and CIN 3 are collectively as high-grade CIN for histologic term and referred to as high-grade squamous intraepithelial lesion in cytological counterpart, (HSIL; including moderate dysplasia, severe dysplasia or carcinoma *in situ* for histologic term) where mitotic and immature cells respectively occur in the middle as well as upper thirds of the epithelium or whole epithelium, based on the Bethesda system 17, 18.

Single nucleotide polymorphism (SNP) is determined if a gene exerts a different allele in the single nucleotide between the members of a species or paired chromosomes in more than 1% of certain population 19. The genetic variants can influence the expressions of gene by having impacts on the promoter area, exon and 3'-untranslated region, and then genetic susceptibilities occur, and thereafter influence the occurrence of diseases and cancers such as oral cancer 19-21. Although some previous studies have investigated the associations of lncRNA *MALAT1* rs3200401, rs619586 and rs1194338 with breast cancer, 22 colorectal cancer 23 and esophageal cancers, 24 to date no research explores the relationships between these SNPs and CC. Therefore, we designed this study to define the relationships between genetic variants of lncRNA *MALAT1* and the occurrence and clinicopathological parameters of CC and patient 5 years survival.

## Materials and methods

### Enrolled female subjects

This study was designed to consecutively recruit 125 patients with invasive cancer and 98 women with precancerous lesions of uterine cervix from the Department of Obstetrics and Gynecology in Chung Shan Medical University Hospital in Taichung, Taiwan, from February 1994 to February 2015. Meanwhile, 325 female individuals with no previous CC and precancerous lesions who received routine healthcare in the outpatient department of the hospital were recruited as the controls. The 125 CC patients were staged based on the 2009 FIGO. The clinicopathological parameters of CC, including stage, pathologic type, cell grading, stromal invasion depth, tumor diameter, parametrium and vagina invasions as well as pelvic lymph node metastasis, were collected. The studied subjects were assessed for the distribution of 3 lncRNA *MALAT1* genetic variants rs3200401, rs619586 and rs1194338*.* However, only those subjects whose 3 genetic variants of lncRNA* MALAT1* could be all determined were included into analysis. Therefore, 116 patients with cervical invasive cancer, 89 patients with cervical precancerous lesions and 268 control women were recruited for the frequencies of the 3 lncRNA *MALAT1* genetic variants. For the patients having CC or precancerous lesions, cervical biopsy was performed under colposcopy supervision and pathological report was returned with final diagnosis of invasive CC or moderate, severe dysplasia or carcinoma *in situ*. These patients were categorized as cervical neoplasia group. Near 90% of CIN 1 has been found to regress to normal. However, high-grade CIN has been revealed to progress into invasive cancer in considerable rate 25, and is therefore regarded as cervical precancerous lesions. The control groups were categorized as enrolling female individuals without self-reported history of cancer of any site and without cervical neoplasias according to the normal cytological report from cervical Papanicolaou smear and further delineated by normal colposcopic report in the general examination. Included patients received the routine treatment protocols, which accord with guidelines provided by National Comprehensive Cancer Network. The Institutional Review Board in the Chung Shan Medical University Hospital approved this research (CSMUH number: CS18208). All participants had informed consents.

### Deoxyribonucleic acid (DNA) extraction from the blood specimens of all participants and selection of lncRNA *MALAT1* genetic variants

Laboratory staff performed venipuncture techniques to obtain blood specimens from all participants. The specimens were collected into Vacutainer tubes mingled with ethylenediaminetetraacetic acid, and immediately stored at 4 °C. Thereafter, DNA was extracted from white blood cells according to previous study 26, 27. The obtained DNA was dissolved in pH 7.8 TE buffer. Then, it was quantified by the measurement of OD260. The OD260/OD280 ratio was determined and the range of 1.8-2.0 conformed to our criteria and was regarded as pure in order to prevent its cross reactivity from the current homologous RNA in the specimens. The final products were refrigerated at -20 °C and applied as templates for the polymerase chain reaction (PCR).

Based on the data of the international HapMap project and the previous research of Zhao et al. 23, three genetic variants of lncRNA* MALAT1* rs3200401, rs619586 and rs1194338 were selected. Furthermore, these SNPs of lncRNA *MALAT1* gene were associated with the progression of the various cancers 28-31. Genotyping of these 3 lncRNA* MALAT1* polymorphisms was determined using the TaqMan SNP Genotyping Assay with an ABI StepOnePlus^TM^ Real-Time PCR System according to previous report 28. The results were further assessed with SDS version 3.0 software (Applied Biosystems, Foster City, CA, USA).

### Statistical analysis

In order to compare the age distribution of the all participants, analysis of variance (ANOVA) was performed and post hoc analysis was further done by Tamhane test. Chi-square or Fisher's tests were performed to assess the association between genotypic frequencies of lncRNA* MALAT1* genetic variants and the incidence of cervical neoplasias. Age had to be adjusted since the age of patients suffering from cervical precancerous lesions was earlier than the age of patients suffering from cervical invasive cancer. The *p* values were defined by chi-square or Fisher's exact tests as well as by logistic or multinomial logistic regression models for age adjustment. The adjusted odds ratios (AORs) with their 95% confidence intervals (CIs) were used to determine the relationships among genotypic frequencies of lncRNA* MALAT1* SNPs and the incidence of cervical neoplasias (including precancerous lesions and invasive cancer) by the logistic and multinomial logistic regression models for controlling age. Chi-square or Fisher's exact tests were performed to relate lncRNA* MALAT1* polymorphisms with clinicopathological parameters including clinical stage, pathologic type, cell grading, cervical stromal invasion depth, tumor diameter, as well as parametrium and vagina invasions and pelvic lymph node metastasis. In univariate analysis, the Kaplan-Meier curve model was applied to assess the significances of lncRNA* MALAT1* polymorphisms and clinicopathological variables for patient survival in relation to survival time, which were associated with 5 years survival of CC patients. The log-rank test was done to distinguish the differences among them. In multivariate analysis, the impacts of lncRNA* MALAT1* polymorphisms and these clinicopathological factors on 5 years survival of these patients were evaluated using the Cox proportional hazard model corresponding survival time. The hazard ratios (HRs) were then defined. The SPSS, version 18.0 and WinPepi Software, version 10.0 were applied for statistical analysis. *P* <0.05 was determined having statistically significant difference.

## Results

There was statistically different age distribution between patients with cervical neoplasm and control women (50.7 ± 13.5 vs. 43.5 ± 9.6, *p*<0.001). Based on the ANOVA with Tamhane test as post hoc analysis, the age distributions were significantly different between patients with CC and patients with precancerous lesions (55.8 ± 12.3 vs. 44.2 ± 12.1, *p*<0.001) as well as between CC patients and control females (55.8 ± 12.3 vs. 43.5 ± 9.6, *p*<0.001). But, there was no significant difference for the age distribution between patients with precancerous lesions and control females (44.2 ± 12.1 vs. 43.5 ± 9.6, *p*= 0.953).

Minor allele frequency of lncRNA* MALAT1* polymorphisms all > 5% in control Taiwanese women. Genotypic distribution of lncRNA* MALAT1* polymorphism rs3200401 met the Hardy-Weinberg equilibrium [χ2 value, 0.806, *p*=0.668; degree of freedom (d.f.)=2] in control women. The distributions of rs619586 and rs1194338 all conformed to the Hardy-Weinberg equilibrium (χ2 value, 1.510, *p*=0.470 and χ2 value, 0.769, *p*=0.681; respectively).

No significant difference was found for the genotypic frequencies of C/C, C/T and T/T in lncRNA* MALAT1* genetic variant rs3200401 between patients with cervical neoplasias and control women (*p*=0.929). The distributions of other lncRNA* MALAT1* genotypic variants, rs619586 and rs1194338 revealed no significant difference between patients with cervical neoplasias and control women (*p*=0.341 and 0.62, respectively; Table 1). Although age was controlled for adjustment, there were no significantly different genotypic frequencies of lncRNA* MALAT1* polymorphisms between patients with cervical neoplasias and control women (*p*=0.995, 0.523, and 0.749, respectively; Table 1).

When cervical neoplasias patients' group was further categorized into subgroups of patients with invasive cancer and those with precancerous lesions, there were still no significantly different in the genotypic frequencies of C/C, C/T and T/T in lncRNA* MALAT1* genetic variant rs3200401 among invasive cancer, precancerous and control subgroups (*p*=0.974; Table 2). It also revealed no significant difference in the genotypic frequencies of other lncRNA* MALAT1* genetic variants, rs619586, and rs1194338 (*p*=0.285 and 0.406, respectively; Table 2). Even after controlling for age, the risks of precancerous lesions and invasive cancer of uterine cervix were not associated with genotypic frequencies of these lncRNA* MALAT1* genetic variants (Table 2).

The associations of lncRNA* MALAT1* genetic variants with clinicopathological parameters of CC, including stage, pathologic type, cell grading, stromal invasion depth, tumor diameter, parametrium and vagina invasions as well as pelvic lymph node metastasis, were further investigated. However, it revealed that no associations were found between genotypic distributions of rs3200401 and clinicopathological variables (Table 3). Similarly, no relationships were found among rs619586 and rs1194338 as well as these parameters (Table 3).

In univariate analysis, C/T+T/T and T/T were not related to 5 years survival in CC patients separately using C/C and C/C+C/T as references in lncRNA *MALAT1* polymorphism rs3200401 (*p*=0.675, HR: 0.80, 95% CI: 0.29-2.25; *p*=0.260, HR: 3.01, 95% CI: 0.40-22.6, respectively; Table 4). Other genetic variants rs619586, and rs1194338 were also not related to 5 years survival in CC patients. However, HRs with worse 5 years survival could be revealed in CC patients with deep stromal invasion (*p*=0.009, HR: 4.00, 95% CI: 1.30-12.28), bigger tumor (*p*=0.011, HR: 3.50, 95% CI: 1.25-9.82), positive parametrium invasion (*p*=0.034, HR: 2.67, 95% CI: 1.03-6.89) and positive pelvic lymph node metastasis (*p*<0.001, HR: 8.11, 95% CI: 2.89-22.78; Table 4).

In multivariate analysis, lncRNA* MALAT1* genetic variants C/T+T/T and T/T were not related to 5 years survival in CC patients separately using C/C and C/C+C/T as references in lncRNA *MALAT1* polymorphism rs3200401 (*p*=0.612, HR: 0.65, 95% CI: 0.12-3.44; *p*=0.409, HR: 3.12, 95% CI: 0.21-46.36, respectively; Table 5). Other genetic variants rs619586 and rs1194338 were also not related to 5 years survival in CC patients. The only independent factor among LncRNA* MALAT1* genetic variants and clinicopathological parameters were positive pelvic lymph node metastasis (*p*=0.001, HR: 10.94, 95% CI: 2.65-45.23; Table 5).

## Discussion

It was revealed that lncRNA* MALAT1* was correlated with gynecological cancers. *MALAT1* may activate cancer cell proliferation and reduce apoptosis by targeting miR-503-5p in ovarian cancer 32, 33; as well as is related to cancer risk and promote cell migration and invasiveness in endometrial cancer 34, 35. Moreover, it has been shown that *MALAT1* may promote cell migration and invasiveness, and epithelial-to-mesenchymal transition in CC 10-12. Han et al. reported that MALAT1/miR-202-3p/periostin axis was associated with the regulation of the cell migration in CC 11. Wang et al. reveled that MALAT1 regulated the cell apoptosis via BRWD1 and PI3K/AKT pathway in CC 12. Cancer susceptibility has been revealed to be correlated with various SNPs in lncRNAs by genome-wide association studies 36, 37. In addition, disease associated genetic variants may be situated in noncoding regions, comprising intergenic, intronic, and regulatory regions 38, 39. LncRNAs expression may be influenced by SNPs occurring in the regulatory regions of lncRNAs, which can enhance or disrupt the binding of transcription factors to DNA 40, 41. LncRNA *MALAT1* genetic variants rs3200401 and rs619586 are two tagSNPs and rs1194338 is situated in the promoter region of *MALAT1.* Because lncRNAs SNPs may influence the gene expression and until now no study investigates the relationships between these *MALAT1* SNPs and uterine CC, this study is conducted to associate their relationships.

In this research, no significant differences occurred in the genotypic frequencies of three lncRNA *MALAT1* genetic variants rs3200401, rs619586 and rs1194338 between Taiwanese women with cervical neoplasias and controls. Even after cervical neoplasia patients' group was categorized into invasive and precancerous subgroups as well as age was adjusted, no significant genotypic frequencies of these *MALAT1* variants were still found among them. To the best of our knowledge, no study investigates the relationships between *MALAT1* polymorphisms and the development of CC. However in ovarian cancer, the heterozygous A/G and the homozygous mutant G/G were related to risk reduction as compared to the wild type AA in *MALAT1* rs619586 42. Hong et al. found that C/T in the codominant model and C/T+T/T in the dominant model in *MALAT1* rs3200401 were related to the elevation of gastric cancer risk in male patients but not in female patients 43. By the contrast, Peng et al. revealed that genotype C/T in *MALAT1* rs3200401 was related to reduction of breast cancer risk in patients who were older than 50 years, as compared to C/C+T/T 22. The genetic variants in *MALAT1* rs3200401 are situated in the *MALAT1* nucleotides 6008-7001, which can be used for the binding sites of serine and arginine rich splicing factor 2 44. *MALAT1* rs3200401 has been proposed that it can interact with serine/arginine-rich proteins and then modulates the alternative splicing of pre-miRNAs. The genetic variation in rs3200401 probably modulates the expression of cancer-associated gene and therefore affects cancer development.

*MALAT1* has been demonstrated to promote cell migration and invasiveness in uterine CC 10, 11. We therefore delineated the associations of lncRNA *MALAT1* polymorphisms rs3200401, rs619586 and rs1194338 with clinopathological variables of CC. However, *MALAT1* SNPs were not found to be related to clinicopathological parameters of CC in this study. In contrast, *MALAT1* rs3200401 has been found to be correlated with histological type of gastric cancer 43. In addition, female patients with hepatocellular carcinoma (HCC) who had the genotypes C/A+A/A in *MALAT1* rs1194338 exhibited a lower risk of vascular invasion and a risk of high Child-Pugh grade (B or C) 30. Moreover, Ji et al. found that *MALAT1* genetic variants rs3200401 and rs619586 were not related to TNM staging and metastasis of HCC in a Southern Chinese population 45.

In univariate and multivariate analyses, lncRNA *MALAT1* SNPs had no significant impact on 5 years survival in CC patients. Increased HRs with worse 5 years survival could be revealed in CC patients with deeper stromal invasion, bigger tumor, positive parametrium invasion and positive pelvic lymph node metastasis. Furthermore, after adjusting these SNPs and clinicopathological factors through multivariate analysis, positive pelvic lymph node metastasis was demonstrated to be the only independently worse predictor of 5 years survival among these variables in CC patients in Taiwanese women. Other studies also corroborate this finding 46, 47, and 5 years survival rate significantly reducing from 85%-90% in positive pelvic lymph node metastasis to 30%-50% in negative lymph node metastasis in CC patients 48. However, it has been revealed that genotypes C/T and C/T +T/T in *MALAT1* rs3200401 were related to reduce the risk of death in non-small-cell lung cancer 49.

To our knowledge, the research may be the first one to investigate the roles of lncRNA *MALAT1* genetic variant*s* rs3200401, rs619586 and rs1194338 in uterine CC. However, this study has some limitations. First, the research was a hospital-based cohort study and occurrence of selection bias was inevitably possible. Second, this study only included the population of central Taiwan and did not recruit residents from other regions, and only contained those subjects whose 3 genetic variants of lncRNA* MALAT1* could be all determined. The enrolled sample size therefore may not be large enough to obtain a statistical significance, especially for precancerous groups, thus restricting the possible subgroup analysis. The external validity of this research may be limited. Third, because the occurrence ages of cervical precancerous lesions and invasive cancer are inherently different, the age distributions of these diseases are different. Therefore, logistic regression models with adjustment were applied to reduce the influence of age. Fourth, female individuals in the control group were enrolled from the outpatient clinic of our hospital for general examination. Because of their conservative attitude, routine examination for human papillomavirus infection (HPV) was not popular. The impact of HPV cannot be included to assess.

## Figures and Tables

**Table 1:** Genetic variant distributions of long noncoding RNA* metastasis-associated lung adenocarcinoma transcript 1* in female patients with cervical neoplasias and control females in Taiwan

Genetic variants	Controls (n =268)	Cervical neoplasias^a^ (n=205)	ORs (95% CIs)	*P* value	AORs (95% CIs)^b^	Adjusted *P* values^b^
**rs3200401**				0.929		0.995
C/C^c^	176	137	1.00		1.00	
C/T	85	62	0.94 (0.63-1.39)	0.748	0.98 (0.65-1.49)	0.939
T/T	7	6	1.10 (0.36-3.35)	0.865	1.04 (0.33-3.23)	0.953
C/C^c^	176	137	1.00	0.792	1.00	0.954
C/T & T/T	92	68	0.95 (0.65-1.40)		0.99 (0.66-1.48)	
C/C & C/T^c^	261	199	1.00	0.836	1.00	0.945
T/T	7	6	1.12 (0.37-3.40)		1.04 (0.34-3.23)	
**rs619586**				0.341		0.523
A/A^c^	230	182	1.00		1.00	
A/G	38	23	0.77 (0.44-1.33)	0.342	0.83 (0.47-1.48)	0.523
G/G	0	0	u.a.	u.a.	u.a.	u.a.
A/A^c^	230	182	1.00	0.342	1.00	0.523
A/G & G/G	38	23	0.77 (0.44-1.33)		0.83 (0.47-1.48)	
A/A & A/G^c^	268	205	1.00	u.a.	1.00	u.a.
G/G	0	0	u.a.		u.a.	
**rs1194338**				0.626		0.749
C/C^c^	112	85	1.00		1.00	
C/A	127	92	0.96 (0.65-1.41)	0.815	1.00 (0.67-1.51)	0.993
A/A	29	28	1.27 (0.71-2.30)	0.425	1.26 (0.67-2.34)	0.473
C/C^c^	112	85	1.00	0.943	1.00	0.805
C/A & A/A	156	120	1.01 (0.70-1.47)		1.05 (0.71-1.55)	
C/C & C/A^c^	239	177	1.00	0.348	1.00	0.447
A/A	29	28	1.30 (0.75-2.27)		1.26 (0.70-2.25)	

Statistical analysis: logistic regression model, chi-square or Fisher's exact tests. ^a^Cervical neoplasias consist of precancerous lesions and invasive cancer of the uterine cervix. ^b^The adjusted *p* values as well as adjusted odds ratios (AORs) and their 95% confident intervals (95% CIs) were defined by logistic regression model after controlling age. ^c^Used as a reference for comparison to determine the odds ratios of other genotypes. u.a., unavailable.

**Table 2:** Genetic variant distributions of long noncoding RNA* metastasis-associated lung adenocarcinoma transcript 1* in female patients with uterine cervical invasive cancer or precancerous lesions and control individuals in Taiwan

Genetic variants	Controls (n =268)	Pre-cancerous lesions (n =89)	Invasive cancer (n =116)	*P* value	AORs (95% CIs)^a^	Ad. *P* values	AORs (95% CIs)^b^	Ad. *P* values
**rs3200401**								
C/C^c^	176	59	78	0.974	1.00		1.00	
C/T	85	28	34		0.99 (0.59-166)	0.967	0.94 (0.55-1.61)	0.831
T/T	7	2	4		0.84 (0.17-4.16)	0.830	1.32 (0.35-4.98)	0.679
C/C^c^	176	59	78	0.956	1.00		1.00	
C/T & T/T	92	30	38		0.98 (0.59-1.62)	0.930	0.98 (0.58-1.64)	0.930
C/C & C/T^c^	261	87	112	0.854	1.00		1.00	
T/T	7	2	4		0.84 (0.17-4.14)	0.832	1.35 (0.36-5.02)	0.657
**rs619586**								
A/A^c^	230	76	106	0.285	1.00		1.00	
A/G	38	13	10		1.04 (0.53-2.06)	0.909	0.62 (0.28-1.38)	0.240
G/G	0	0	0		u.a.	u.a.	u.a.	u.a.
A/A^c^	230	76	106	0.285	1.00		1.00	
A/G & G/G	38	13	10		1.04 (0.53-2.06)	0.909	0.62 (0.28-1.38)	0.240
A/A & A/G^c^	268	89	116	u.a.	1.00		1.00	
G/G	0	0	0		u.a.	u.a.	u.a.	u.a.
**rs1194338**								
C/C^c^	112	33	52	0.406	1.00		1.00	
C/A	127	46	46		1.23 (0.74-2.06)	0.425	0.79 (0.47-1.33)	0.375
A/A	29	10	18		1.17 (0.52-2.66)	0.701	1.26 (0.59-2.70)	0.548
C/C^c^	112	33	52	0.535	1.00		1.00	
C/A & A/A	156	56	64		1.22 (0.75-2.00)	0.427	0.88 (0.54-1.44)	0.607
C/C & C/A^c^	239	79	98	0.416	1.00		1.00	
A/A	29	10	18		1.04 (0.49-2.24)	0.911	1.42 (0.70-2.90)	0.335
A/A	0	0	0		u.a.	u.a.	u.a.	u.a.

Statistical analysis: multinomial logistic regression models, chi-square or Fisher's exact tests. ^a^Adjusted *p* values and adjusted odds ratios with their 95% CIs were defined by multinomial logistic regression models after controlling for age between patients with uterine cervical precancerous lesions and control females. ^b^Adjusted *p* values and adjusted odds ratios with their 95% CIs were defined by multinomial logistic regression models after controlling for age between patients with uterine cervical invasive cancer and control females. ^c^Used as a reference for comparison to assess the odds ratios of other genotypes. AORs, adjusted odds ratios; 95% CIs, 95% confidence intervals; Ad. *p*, adjusted *p*; u.a., unavailable.

**Table 3:** Associations between genotypic distributions of long noncoding RNA *metastasis-associated lung adenocarcinoma transcript 1* and clinicopathological parameters of the patients with cervical invasive cancer

Parameters^a^	rs3200401				rs619586				rs1194338			
	CC^b^	CT/TT	CC/CT^b^	TT	AA^b^	AG/GG	AA/AG^b^	GG	CC^b^	CA/AA	CC/CA^b^	AA
**Clinical stage**												
stage I^b^	45	20	64	1	57	8	65	0	27	38	58	7
≥ stage II	33	17	47	3	48	2	50	0	25	25	40	10
*P* value	0.713		0.316		0.183		u.a.		0.366		0.167	
**Pathologic type**												
squamous cell carcinoma^b^	67	35	99	3	93	9	102	0	44	58	85	17
adenocarcinoma	11	3	13	1	13	1	14	0	8	6	13	1
*P* value	0.544		0.407		1.000		u.a.		0.323		0.693	
**Cell grading**												
well (grade 1)^b^	13	6	19	0	18	1	19	0	8	11	17	2
moderate & poor (grades 2/3)	65	32	93	4	88	9	97	0	44	53	81	16
*P* value	0.905		1.000		1.000		u.a.		0.794		0.733	
**Stromal invasion depth**												
≤10 mm^b^	41	17	57	1	50	8	58	0	25	33	50	8
>10 mm	32	18	48	2	48	2	50	0	24	26	43	7
*P* value	0.459		0.595		0.103		u.a.		0.610		0.975	
**Tumor diameter**												
≤4 cm^b^	44	20	63	1	56	8	64	0	26	38	56	8
>4 cm	33	17	47	3	48	2	50	0	26	24	41	9
*P* value	0.756		0.318		0.182		u.a.		0.226		0.413	
**Parametrium**												
no invasion^b^	51	21	71	1	64	8	72	0	31	41	64	8
invasion	26	16	39	3	40	2	42	0	21	21	33	9
*P* value	0.326		0.141		0.320		u.a.		0.473		0.136	
**Vagina**												
no invasion^b^	51	21	71	1	64	8	72	0	31	41	63	9
invasion	26	16	39	3	40	2	42	0	21	21	34	8
*P* value	0.326		0.141		0.320		u.a.		0.473		0.344	
**Pelvic lymph node**												
no metastasis^b^	58	26	81	3	76	8	84	0	38	46	74	10
metastasis	19	11	29	1	28	2	30	0	14	16	23	7
*P* value	0.566		1.000		1.000		u.a.		0.893		0.144	

Statistical analyses: chi-square or Fisher's exact tests. ^a^Clinicopathological data of some cases could not be obtained from the patients with cervical invasive cancer due to incomplete medical charts or records. ^b^As a reference: u.a., unavailable.

**Table 4:** Univariate analysis of genetic variants of long noncoding RNA* metastasis-associated lung adenocarcinoma transcript 1* and clinicopathological variables for 5 years survival in cervical cancer patients

Variables^a^	5 years survival		*P* value	HR (95% CIs)^c^
	+	-		
**rs3200401**				
C/C^b^	63	13	0.675	1.00
C/T+T/T	32	5		0.80 (0.29-2.25)
C/C+C/T^b^	93	17	0.260	1.00
T/T	2	1		3.01 (0.40-22.6)
**rs619586**				
A/A^b^	87	16	0.884	1.00
A/G+G/G	8	2		1.12 (0.26-4.86)
A/A+A/G^b^	95	18	u.a.	1.00
G/G	0	0		u.a.
**rs1194338**				
C/C^b^	42	10	0.409	1.00
C/A+A/A	53	8		0.68 (0.27-1.72)
C/C+C/A^b^	82	15	0.747	1.00
A/A	13	3		1.23 (0.36-4.23)
**Clinical stage**				
stage I^b^	56	6	0.046	1.00
≥stage II	38	12		2.61 (0.98-6.95)
**Pathologic type**				
squamous cell carcinoma^b^	86	14	0.162	1.00
adenocarcinoma	9	4		2.16 (0.71-6.58)
**Cell grading**				
well (grade 1)^b^	15	3	0.942	1.00
moderate & poor (grades 2/3)	80	15		1.05 (0.30-3.62)
**Stromal invasion depth**				
≤ 10 mm^b^	51	4	0.009	1.00
> 10 mm	37	13		4.00 (1.30-12.28)
**Tumor diameter**				
≤ 4 cm^b^	56	5	0.011	1.00
> 4 cm	37	13		3.50 (1.25-9.82)
**Parametrium**				
no invasion^b^	62	7	0.034	1.00
invasion	31	11		2.67 (1.03-6.89)
**Vagina**				
no invasion^b^	60	9	0.189	1.00
invasion	33	9		1.84 (0.73-4.64)
**Pelvic lymph node**				
no metastasis^b^	76	5	<0.001	1.00
metastasis	17	13		8.11 (2.89-22.78)

Statistical analyses: Kaplan-Meier curve model. ^a^Clinicopathological data of some cases could not be obtained from the patients with cervical invasive cancer due to incomplete records of medical chart. ^b^As a reference. ^c^HR, hazard ratio and 95% CI, 95% confidence interval for lncRNA* MALAT1* genetic variants rs3200401, rs619586 and rs1194338 and clinicopathological factors, compared to their respective controls. Survival: +, survival, -, mortality; u.a., unavailable.

**Table 5:** Multivariate analysis of the relationships among long noncoding RNA* metastasis-associated lung adenocarcinoma transcript 1* variants and clinicopatholgical variables and 5 years survival of cervical cancer patients

Variables	5 years survival	
	*P* value	HR & 95% CI^b^
**lncRNA* MALAT1* genetic variants**		
**rs3200401**		
C/T+T/T vs. C/C^a^	0.612	0.65 (0.12-3.44)
T/T vs. C/C & C/T^a^	0.409	3.12 (0.21-46.36)
**rs619586**		
A/G+G/G vs. A/A^a^	0.344	2.21 (0.43-11.42)
G/G vs. A/A+A/G^ a^	u.a.	u.a.
**rs1194338**		
C/A+A/A vs. C/C^a^	0.289	0.43 (0.09-2.04)
A/A vs. C/C+C/A^a^	0.569	1.75 (0.26-12.04)
**Clinicopathological characteristics**		
**Pelvic lymph node**		
metastasis vs. no metastasis^a^	0.001	10.94 (2.65-45.23)

Statistical analyses: Cox proportional hazard model; ^a^As a comparison reference; ^b^HR, hazard ratio and 95% CI, 95% confidence interval for lncRNA* MALAT1* genetic variants rs3200401, rs619586 and rs1194338 and clinicopathological factors, compared to their respective controls. lncRNA *MALAT1*, long noncoding RNA* metastasis-associated lung adenocarcinoma transcript 1*; u.a., unavailable.

## References

[1] Rinn JL, Kertesz M, Wang JK, Squazzo SL, Xu X, Brugmann SA (2007). Functional demarcation of active and silent chromatin domains in human HOX loci by noncoding RNAs. Cell.

[2] Denzler R, Agarwal V, Stefano J, Bartel DP, Stoffel M (2014). Assessing the ceRNA hypothesis with quantitative measurements of miRNA and target abundance. Mol Cell.

[3] Caceres-Duran MA, Ribeiro-Dos-Santos A, Vidal AF (2020). Roles and Mechanisms of the Long Noncoding RNAs in Cervical Cancer. Int J Mol Sci.

[4] Valenti G, Vitale SG, Tropea A, Biondi A, Lagana AS (2017). Tumor markers of uterine cervical cancer: a new scenario to guide surgical practice?. Updates Surg.

[5] Zhang X, Hamblin MH, Yin KJ (2017). The long noncoding RNA Malat1: Its physiological and pathophysiological functions. RNA Biol.

[6] Hutchinson JN, Ensminger AW, Clemson CM, Lynch CR, Lawrence JB, Chess A (2007). A screen for nuclear transcripts identifies two linked noncoding RNAs associated with SC35 splicing domains. BMC Genomics.

[7] Wang C, Zhang Q, Hu Y, Zhu J, Yang J (2019). Emerging role of long non-coding RNA MALAT1 in predicting clinical outcomes of patients with digestive system malignancies: A meta-analysis. Oncol Lett.

[8] Li ZX, Zhu QN, Zhang HB, Hu Y, Wang G, Zhu YS (2018). MALAT1: a potential biomarker in cancer. Cancer Manag Res.

[9] Arun G, Spector DL (2019). MALAT1 long non-coding RNA and breast cancer. RNA Biol.

[10] Yang L, Bai HS, Deng Y, Fan L (2015). High MALAT1 expression predicts a poor prognosis of cervical cancer and promotes cancer cell growth and invasion. Eur Rev Med Pharmacol Sci.

[11] Han X, Wang Q, Wang Y, Hu B, Dong X, Zhang H (2019). Long non-coding RNA metastasis-associated lung adenocarcinoma transcript 1/microRNA-202-3p/periostin axis modulates invasion and epithelial-mesenchymal transition in human cervical cancer. Journal of cellular physiology.

[12] Wang N, Hou MS, Zhan Y, Shen XB, Xue HY (2018). MALAT1 promotes cisplatin resistance in cervical cancer by activating the PI3K/AKT pathway. European review for medical and pharmacological sciences.

[13] Sung H, Ferlay J, Siegel RL, Laversanne M, Soerjomataram I, Jemal A (2021). Global Cancer Statistics 2020: GLOBOCAN Estimates of Incidence and Mortality Worldwide for 36 Cancers in 185 Countries. CA: a cancer journal for clinicians.

[14] Portnoy A, Clark S, Ozawa S, Jit M (2020). The impact of vaccination on gender equity: conceptual framework and human papillomavirus (HPV) vaccine case study. Int J Equity Health.

[15] Hsin MC, Hsieh YH, Wang PH, Ko JL, Hsin IL, Yang SF (2017). Hispolon suppresses metastasis via autophagic degradation of cathepsin S in cervical cancer cells. Cell Death Dis.

[16] Nasiell K, Roger V, Nasiell M (1986). Behavior of mild cervical dysplasia during long-term follow-up. Obstet Gynecol.

[17] Petignat P, Roy M (2007). Diagnosis and management of cervical cancer. BMJ.

[18] Khan MJ, Castle PE, Lorincz AT, Wacholder S, Sherman M, Scott DR (2005). The elevated 10-year risk of cervical precancer and cancer in women with human papillomavirus (HPV) type 16 or 18 and the possible utility of type-specific HPV testing in clinical practice. J Natl Cancer Inst.

[19] Shastry BS (2009). SNPs: impact on gene function and phenotype. Methods Mol Biol.

[20] Hua KT, Liu YF, Hsu CL, Cheng TY, Yang CY, Chang JS (2017). 3'UTR polymorphisms of carbonic anhydrase IX determine the miR-34a targeting efficiency and prognosis of hepatocellular carcinoma. Scientific reports.

[21] Su CW, Chien MH, Lin CW, Chen MK, Chow JM, Chuang CY (2018). Associations of genetic variations of the endothelial nitric oxide synthase gene and environmental carcinogens with oral cancer susceptibility and development. Nitric oxide: biology and chemistry.

[22] Peng R, Luo C, Guo Q, Cao J, Yang Q, Dong K (2018). Association analyses of genetic variants in long non-coding RNA MALAT1 with breast cancer susceptibility and mRNA expression of MALAT1 in Chinese Han population. Gene.

[23] Zhao K, Jin S, Wei B, Cao S, Xiong Z (2018). Association study of genetic variation of lncRNA MALAT1 with carcinogenesis of colorectal cancer. Cancer Manag Res.

[24] Qu Y, Shao N, Yang W, Wang J, Cheng Y (2019). Association of polymorphisms in MALAT1 with the risk of esophageal squamous cell carcinoma in a Chinese population. Onco Targets Ther.

[25] Martin CM, O'Leary JJ (2011). Histology of cervical intraepithelial neoplasia and the role of biomarkers. Best Pract Res Clin Obstet Gynaecol.

[26] Sun YH, Chou YH, Ou CC, Ng SC, Shen HP, Lee YC (2020). Investigation of metastasis-associated in colon cancer-1 genetic variants in the development and clinicopathologcial characteristics of uterine cervical cancer in Taiwanese women. International journal of medical sciences.

[27] Hsiao PC, Chen MK, Su SC, Ueng KC, Chen YC, Hsieh YH (2010). Hypoxia inducible factor-1alpha gene polymorphism G1790A and its interaction with tobacco and alcohol consumptions increase susceptibility to hepatocellular carcinoma. Journal of surgical oncology.

[28] Ding YF, Wen YC, Chuang CY, Lin CW, Yang YC, Liu YF (2021). Combined Impacts of Genetic Variants of Long Non-Coding RNA MALAT1 and the Environmental Carcinogen on the Susceptibility to and Progression of Oral Squamous Cell Carcinoma. Frontiers in oncology.

[29] Hu JC, Wang SS, Chou YE, Chiu KY, Li JR, Chen CS (2021). Associations between LncRNA MALAT1 Polymorphisms and Lymph Node Metastasis in Prostate Cancer. Diagnostics (Basel, Switzerland).

[30] Yuan LT, Chang JH, Lee HL, Yang YC, Su SC, Lin CL (2019). Genetic Variants of lncRNA MALAT1 Exert Diverse Impacts on the Risk and Clinicopathologic Characteristics of Patients with Hepatocellular Carcinoma. Journal of clinical medicine.

[31] Wu S, Sun H, Wang Y, Yang X, Meng Q, Yang H (2019). MALAT1 rs664589 Polymorphism Inhibits Binding to miR-194-5p, Contributing to Colorectal Cancer Risk, Growth, and Metastasis. Cancer research.

[32] Hosseini ES, Meryet-Figuiere M, Sabzalipoor H, Kashani HH, Nikzad H, Asemi Z (2017). Dysregulated expression of long noncoding RNAs in gynecologic cancers. Mol Cancer.

[33] Sun Q, Li Q, Xie F (2019). LncRNA-MALAT1 regulates proliferation and apoptosis of ovarian cancer cells by targeting miR-503-5p. Onco Targets Ther.

[34] Chen G, Zhang M, Liang Z, Chen S, Chen F, Zhu J (2020). Association of polymorphisms in MALAT1 with the risk of endometrial cancer in Southern Chinese women. J Clin Lab Anal.

[35] Zhao Y, Yang Y, Trovik J, Sun K, Zhou L, Jiang P (2014). A novel wnt regulatory axis in endometrioid endometrial cancer. Cancer Res.

[36] Cheetham SW, Gruhl F, Mattick JS, Dinger ME (2013). Long noncoding RNAs and the genetics of cancer. Br J Cancer.

[37] Chen G, Qiu C, Zhang Q, Liu B, Cui Q (2013). Genome-wide analysis of human SNPs at long intergenic noncoding RNAs. Hum Mutat.

[38] Freedman ML, Monteiro AN, Gayther SA, Coetzee GA, Risch A, Plass C (2011). Principles for the post-GWAS functional characterization of cancer risk loci. Nat Genet.

[39] Hindorff LA, Sethupathy P, Junkins HA, Ramos EM, Mehta JP, Collins FS (2009). Potential etiologic and functional implications of genome-wide association loci for human diseases and traits. Proc Natl Acad Sci U S A.

[40] Guo H, Ahmed M, Zhang F, Yao CQ, Li S, Liang Y (2016). Modulation of long noncoding RNAs by risk SNPs underlying genetic predispositions to prostate cancer. Nat Genet.

[41] Huang Q, Whitington T, Gao P, Lindberg JF, Yang Y, Sun J (2014). A prostate cancer susceptibility allele at 6q22 increases RFX6 expression by modulating HOXB13 chromatin binding. Nat Genet.

[42] Huang X, Zhang W, Shao Z (2018). Association between long non-coding RNA polymorphisms and cancer risk: a meta-analysis. Biosci Rep.

[43] Hong JH, Jin EH, Chang IA, Kang H, Lee SI, Sung JK (2020). Association of long noncoding RNA MALAT1 polymorphisms with gastric cancer risk in Korean individuals. Mol Genet Genomic Med.

[44] Miyagawa R, Tano K, Mizuno R, Nakamura Y, Ijiri K, Rakwal R (2012). Identification of cis- and trans-acting factors involved in the localization of MALAT-1 noncoding RNA to nuclear speckles. RNA.

[45] Ji X, Zhang J, Liu L, Lin Z, Pi L, Lin Z (2019). Association of tagSNPs at lncRNA MALAT-1 with HCC Susceptibility in a Southern Chinese Population. Sci Rep.

[46] Kamura T, Tsukamoto N, Tsuruchi N, Saito T, Matsuyama T, Akazawa K (1992). Multivariate analysis of the histopathologic prognostic factors of cervical cancer in patients undergoing radical hysterectomy. Cancer.

[47] Choi KH, Kim JY, Lee DS, Lee YH, Lee SW, Sung S (2018). Clinical impact of boost irradiation to pelvic lymph node in uterine cervical cancer treated with definitive chemoradiotherapy. Medicine (Baltimore).

[48] Monk BJ, Cha DS, Walker JL, Burger RA, Ramsinghani NS, Manetta A (1994). Extent of disease as an indication for pelvic radiation following radical hysterectomy and bilateral pelvic lymph node dissection in the treatment of stage IB and IIA cervical carcinoma. Gynecol Oncol.

[49] Wang JZ, Xiang JJ, Wu LG, Bai YS, Chen ZW, Yin XQ (2017). A genetic variant in long non-coding RNA MALAT1 associated with survival outcome among patients with advanced lung adenocarcinoma: a survival cohort analysis. BMC Cancer.

